# Genetic, clinical, lifestyle and sociodemographic risk factors for head and neck cancer: A UK Biobank study

**DOI:** 10.1371/journal.pone.0318889

**Published:** 2025-04-04

**Authors:** Lisa Tuomi, Toshima Z. Parris, Araz Rawshani, Erik Andersson, Alina Orozco, Caterina Finizia

**Affiliations:** 1 Department of Health and Rehabilitation, Institute of Neuroscience and Physiology, Sahlgrenska Academy, University of Gothenburg, Gothenburg, Sweden; 2 Department of Otorhinolaryngology—Head and Neck Surgery, Region Västra Götaland, Sahlgrenska University Hospital, Gothenburg, Sweden; 3 Department of Oncology, Institute of Clinical Sciences, Sahlgrenska Academy, University of Gothenburg, Gothenburg, Sweden; 4 Sahlgrenska Center for Cancer Research, Sahlgrenska Academy, University of Gothenburg, Gothenburg, Sweden; 5 Department of Molecular and Clinical Medicine, Institute of Medicine, Sahlgrenska Academy, University of Gothenburg, Gothenburg, Sweden; 6 Wallenberg Laboratory for Cardiovascular and Metabolic Research, Institute of Medicine, Sahlgrenska Academy, University of Gothenburg, Gothenburg, Sweden; 7 Bioinformatics and Data Centre at the Sahlgrenska Academy and Clinical Genomics Gothenburg at SciLifeLab, Gothenburg, Sweden; 8 Department of Otorhinolaryngology—Head and Neck Surgery, Institute of Clinical Sciences, Sahlgrenska Academy, University of Gothenburg, Gothenburg, Sweden; Far Eastern Memorial Hospital, TAIWAN

## Abstract

**Introduction:**

Despite a steady decline in tobacco smoking, head and neck cancer (HNC) incidence rates are on the rise. Therefore, novel risk factors for HNC are needed to identify at-risk patients at an early stage. Here, we used genetic, clinical, lifestyle, and sociodemographic data from UK Biobank (UKB) to evaluate the relative importance of known risk factors for HNC and identify novel predictors of HNC risk.

**Methods:**

All participants in the UKB between 2006 and 2021 were stratified into HNC cases and controls at baseline (cases: n =  534; controls: n =  501833) or during follow-up (cases: n =  1587; controls: n =  500246). A cross-sectional description of risk factors (clinical characteristics, lifestyle and sociodemographic) for HNC at baseline was performed, followed by multivariate Cox regression analysis (adjusted for age and sex) and gradient boosting machine learning to determine the relative importance of predictors (phenotypic predictors and SNPs) of HNC development after baseline.

**Results:**

In addition to known risk factors for HNC (age, male sex, smoking and alcohol consumption habits, occupation), we show that smoking cessation at ≤ 40 years of age is the strongest predictor of HNC risk. Although SNPs may play a role in HNC development, a predictive model containing phenotypic variables and SNPs (C-index 0.75) did not significantly outperform a model containing the phenotypic predictors alone (C-index 0.73).

**Conclusion:**

Taken together, this study demonstrates that phenotypic variables such as past tobacco smoking habits, occupation, facial pain, education, pulmonary function, and anthropometric measures can be used to predict HNC risk.

## Introduction

Head and neck cancer (HNC) is a collective term for malignant tumors of the nasal cavity, oral cavity, pharynx, and larynx. HNC accounts for approximately 660,000 new cases and 325,000 deaths each year, and is now the seventh most common cancer type worldwide [1,2]. Approximately 50-75% of patients with HNC are diagnosed at an advanced stage, which has a negative effect on quality of life and survival [3]. Incidence rates for HNC are on the rise, with reports predicting a 30% increase by 2030 [4–7], primarily attributed to the rising number of oropharyngeal cancer cases infected with human papillomavirus (HPV; 50-70% in USA and UK) [8]. Other known risk factors for HNC are male sex, age, smoking and alcohol consumption, and betel quid chewing, which is prevalent in Southeast Asia and the Asia-Pacific region.

Recent efforts have been made to identify novel genetic (somatic and germline mutations) and potentially modifiable risk factors that can be used to develop risk prediction models for HNC. In 2015, The Cancer Genome Atlas (TCGA) used whole-exome sequencing to profile head and neck squamous cell carcinomas (HNSCC), thereby demonstrating that HPV-positive HNSCCs are characterized by *PIK3CA* mutations, *TRAF3* loss and *E2F1* gene amplification, while smoking-related HNSCCs are associated with loss-of-function *TP53* mutations and *CDKN2A* inactivation [9]. Numerous large-scale genome-wide association studies (GWAS) have also identified single-nucleotide polymorphisms (SNPs) associated with HPV, metabolic traits, and treatment outcomes in patients with head and neck cancer [10–13]. A review by Smith and colleagues demonstrated that relatively few risk prediction models (n =  14) have been developed for HNC, with only 3 models achieving high performance (i.e., area under the receiver operating characteristic curve exceeding 0.8) [14–17]. These studies used logistic regression and relatively similar predictors (e.g., age, sex, smoking, alcohol use). However, none of these high-performance models included genetic predictors and only 1/8 lower quality studies used genetic data in their risk model [18]. However, these traditional statistical approaches may have limitations when dealing with high-dimensional data and complex interactions among predictors, such as genetic and phenotypic variables. Machine learning (ML) techniques offer several advantages over traditional methods by handling non-linear relationships, feature interactions, and large-scale datasets more effectively. In other cancers, such as breast, prostate, and lung cancer, ML models have demonstrated superior performance in identifying novel predictors, improving risk stratification, and integrating diverse datasets including genetic, phenotypic, and lifestyle factors [19–21].

To assess the relative importance of known risk factors for HNC and identify novel predictors (e.g., SNPs, lifestyle, sociodemographic variables), we studied 502,367 participants enrolled in UK Biobank (UKB) between 2006 and 2021.

## Materials and methods

### Study design and participants

All procedures were performed in accordance with the Declaration of Helsinki and approved by the Swedish Ethical Review Authority (registration number 2023-02503-02). Furthermore, the study was conducted in accordance with the UK Biobank’s research ethics approval (originally 2011, renewed 2021, IRAS Project ID: 299116) and using the UK Biobank Resource under Application Number 76314. A requirement to become a participant of UKB is that written informed consent is given regarding the use of their medical records in scientific research, given that personal identifiers are excluded from data records. All participants enrolled in the UKB up to November 2022 were stratified into HNC cases and controls and subset into the following three study populations: (1) baseline population (no exclusions applied, n =  502367), (2) incident cases (excluding baseline cases of HNC, n =  501833), and (3) cumulative incidence (all cases, n =  502367). HNC at baseline (n =  534) was defined as any HNC diagnosis before the baseline visit in the UKB. Incident cases (n =  1587) were defined as cases diagnosed after baseline examination in the UKB until the end of follow-up on April 26, 2021. Cumulative incidence (n =  2121) was defined as any HNC diagnosis from birth to the end of follow-up.

HNC diagnoses were assessed using International Classification of Diseases version 10 (ICD-10) codes (C00-C14 [malignant neoplasms of lip, oral cavity and pharynx], and C30-C32 [malignant neoplasms of nasal cavity and middle ear, accessory sinuses, and larynx]) available in the Hospital Episode Statistics Admitted Patient Care for England (HESAPC), Patient Episode Database for Wales Admitted Patient Care (PEDWAPC), and Scottish Morbidity Records (SMR). The participants were followed until the date of death, HNC diagnosis or censoring ended on April 26, 2021. The data was initially accessed through DNAnexus on 04/10/2021.

### Candidate predictors of HNC

To identify novel predictors of HNC and reassess previously established associations with the disease, a broad spectrum of candidate predictors of HNC were included in the study. In UKB, data on a wide range of exposures and health outcomes were collected using digital questionnaires and/or interviews. Phenotypic predictors were stratified into eight categories: (1) sociodemographic characteristics (e.g., age, sex, ethnicity, household income, education), (2) anthropometry (e.g., body mass index [BMI]), (3) cardiopulmonary function (e.g., peak expiratory flow [PEF]), (4) coexisting conditions (e.g., overall health rating, hypertension, relevant pain localizations), (5) diet (e.g., major dietary changes), (6) exercise (e.g., physical activity), (7) occupational variables (e.g., employment status, job type), and (8) tobacco and alcohol consumption habits (e.g., alcohol intake, tobacco smoking habits). The NHGRI-EBI GWAS Catalog [22,23] was used to compile SNPs associated with HNC by querying for the following search terms, i.e., “head and neck malignant neoplasia”, “pharynx cancer”, “head and neck squamous cell carcinoma”, and “oral cavity cancer”. This analysis resulted in the identification of 125 unique SNPs from 10 studies (S1 Table) [10,12,13,24–30]. Genotyping data for the identified GWAS SNPs, that were available from UK Biobank (25/125, 20%), were retrieved. The genotypes were labeled as 0 =  major allele homozygous, 1 =  heterozygous, and 2 =  minor allele homozygous. The genotype frequency, i.e., the percentage of individuals in each group (HNC incident cases vs. controls) with a specific genotype, was calculated for HNC incident cases and controls. The phenotypic predictors included in the study are listed in S2–S9 Tables. Predictors with more than 90% missingness (e.g., “Former alcohol drinker” [91.8%] and “Bipolar disorder status” [99.6%]) were excluded from the analysis to minimize bias that is introduced with little impact on statistical power, aligning with standard practices.

### Outcome assessment

**Fig 1:**
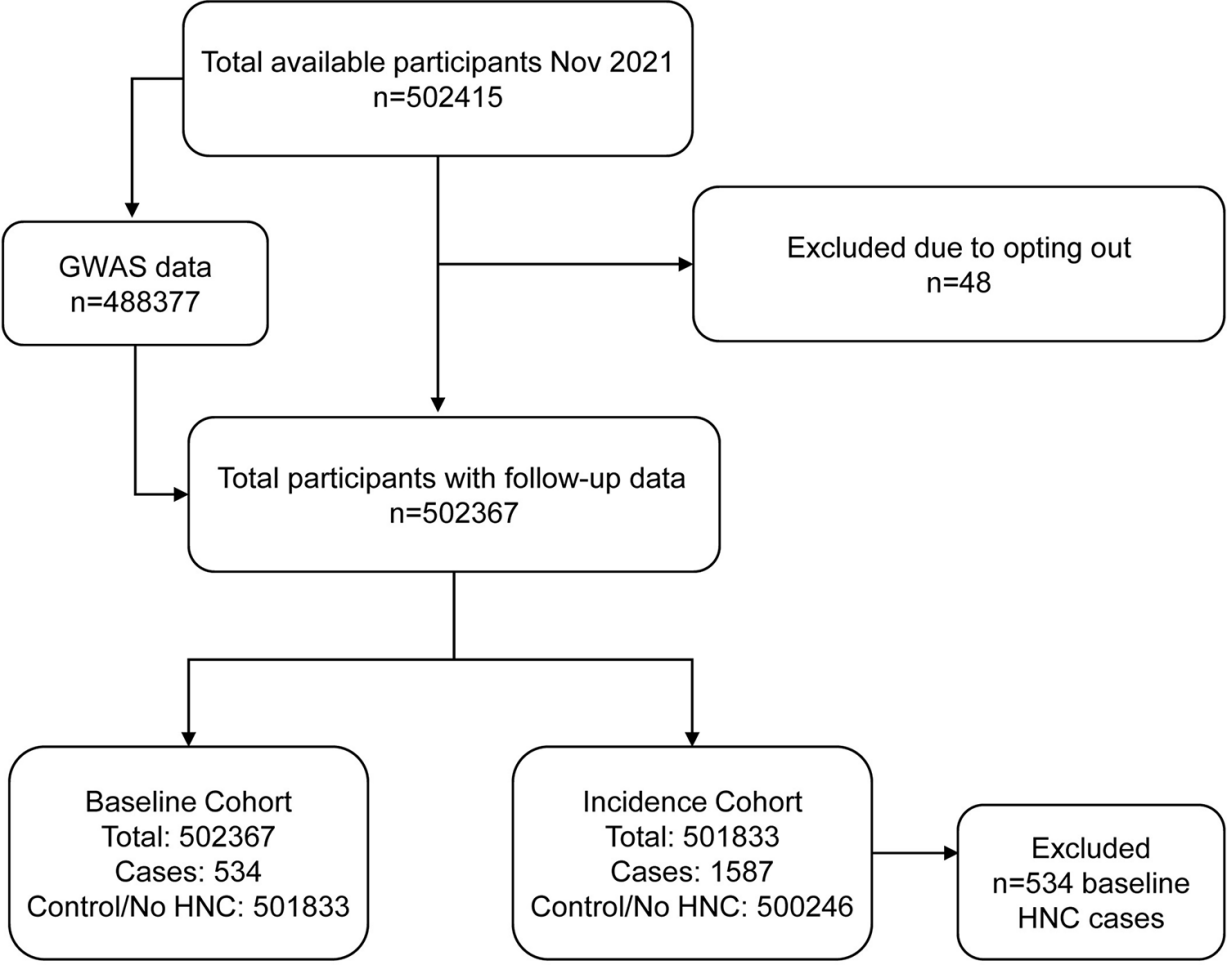
Study workflow.

Three separate analyses of HNC risk were conducted, i.e., (1) a cross-sectional assessment of predictors of HNC at baseline using phenotypic predictors, (2) prospective assessment of HNC risk using phenotypic predictors, and (3) cumulative (lifetime) risk of HNC using genetic predictors. The study workflow is outlined in Fig 1. For the cross-sectional assessment of HNC risk (analysis 1), we used baseline data in the UKB. Individuals with a diagnosis of HNC at baseline were classified as cases (n =  534) and the remaining participants as controls (n =  501833). For the prospective assessment of HNC risk (analysis 2), we used an array of phenotypic predictors (i.e., sociodemography, anthropometry, cardiopulmonary function, coexisting conditions, diet, exercise, occupational variables, and tobacco/alcohol consumption habits), including only participants without a history of HNC at baseline. Any HNC occurring during follow-up was considered as an event. The analysis included all individuals enrolled in the UKB, which were followed to HNC, death or end of follow-up, whichever came first. Of the 501,833 participants that had data up to the end of follow-up, 1,587 participants developed HNC after their baseline visit. To study the association between the SNPs and lifetime risk of HNC (analysis 3), we used all participants in the UKB (n =  502367) to study whether genetic variants that have previously been associated with HNC (according to GWAS Catalog) could predict the development of HNC.

### Statistical analysis

Statistical analyses were performed on the Research Analysis Platform provided by UK Biobank with R (version 4.1.2). TableOne (version 0.13.2) was used to generate descriptive statistics. All p-values were two-sided and *P* <  0.001 was considered statistically significant due to test multiplicity [31]. Participant characteristics were described using the mean and standard deviation (SD) or frequencies (percentages) for categorical variables and median (interquartile range [IQR]) for non-normally distributed continuous variables. Chi-square tests were used for categorical variables and t-tests for continuous variables.

A gradient boosting algorithm (GBM, gradient boosting machine) was used in mlr3proba (version 0.5.2) to study all candidate predictors of HNC. GBM is a prediction model that allows for the calculation of the relative variable importance of each predictor. GBM models were generated using the phenotypic predictors (phenotype model), SNPs (SNP model), and a combined model (phenotype and SNPs). The relative variable importance was also used to select variables for a Cox proportional hazards model to study the association between the most important predictors and risk of HNC, adjusting for age and sex. In order to estimate the extent to which HNC could be predicted using our data, Cox regression models were constructed including the following predictors: 1) all phenotype variables, 2) all SNP variables, 3) all phenotype and SNP variables, 4) top 10 phenotype variables (identified by GBM), 5) top 20 phenotype variables, 6) top 30 phenotype variables, 7) top 40 phenotype variables, 8) top 50 phenotype variables, 9) all SNPs and top 10 phenotypes, 10) all SNPs and top 20 phenotypes, 11) all SNPs and top 30 phenotypes, 12) all SNPs and top 40 phenotypes, and 13) all SNPs and top 50 phenotypes. The predictive performance of each Cox regression model was assessed using concordance index (C-index).

## Results

### Baseline characteristics.

A total of 502,367 participants from the UK Biobank were included in the study, of which 534 (0.11%) had a diagnosis of HNC at baseline. The median age at baseline was 61 years (IQR 54, 64) for patients with HNC compared to 58 years (IQR 50, 63) in the controls (Table 1 and S2–S9 Tables). HNC at baseline was more common in men (68%; *P* <  0.001). Furthermore, patients with HNC were more likely to have a poor or fair overall health rating, as well as lower BMI with lower body fat percentage. Nevertheless, patients with HNC engaged in physical activities less often. Compared to the controls, HNC at baseline was more frequently associated with long standing illness/disability, more medications taken, high blood pressure, greater hand grip strength, pain during the past month, lower household income, and lower educational attainment.

**Table 1:** Patient characteristics associated with head and neck cancer, UK Biobank 2006-2021.

	Baseline				Incidence			
Characteristic	Overall (n = 502367)	No HNC (n = 501833)	HNC (n = 534)	p	Overall (n = 501833)	No HNC (n = 500246)	HNC (n = 1587)	p
**Age at recruitment, median [IQR]**	58.0 [50.0, 63.0]	58.0 [50.0, 63.0]	61.0 [54.0, 64.0]	<0.001	58.0 [50.0, 63.0]	58.0 [50.0, 63.0]	60.0 [54.0, 64.0]	<0.001
**Sex**				<0.001				<0.001
Male	229066 (45.6)	228703 (45.6)	363 (68.0)		228703 (45.6)	227624 (45.5)	1079 (68.0)	
**Ethnic background, n (%)**				0.526				0.004
White	472615 (94.4)	472101 (94.4)	514 (96.4)		472101 (94.4)	470582 (94.4)	1519 (96.1)	
**Average total household income before tax, n (%)**				<0.001				<0.001
Less than £18,000	97176 (22.5)	97010 (22.5)	166 (36.4)		97010 (22.5)	96569 (22.5)	441 (32.1)	
**Qualifications, n (%)**				<0.001				<0.001
College or University degree	161104 (32.1)	160989 (32.1)	115 (21.5)		160989 (32.1)	160612 (32.1)	377 (23.8)	
**Current employment status, n (%)**				<0.001				<0.001
Unable to work because of sickness or disability	16822 (3.3)	16750 (3.3)	72 (13.5)		16750 (3.3)	16583 (3.3)	167 (10.5)	
**Job code at visit, n (%)**				<0.001				<0.001
Skilled Trades Occupations	23463 (7.7)	23429 (7.7)	34 (13.6)		23429 (7.7)	23336 (7.7)	93 (11.6)	
**Job involves heavy manual or physical work, n (%)**				<0.001				<0.001
Never, rarely	186293 (37.1)	186162 (37.1)	131 (24.5)		186162 (37.1)	185711 (37.1)	451 (28.4)	
**Job involves mainly walking or standing, n (%)**				<0.001				<0.001
Never, rarely	100925 (20.1)	100857 (20.1)	68 (12.7)		100857 (20.1)	100613 (20.1)	244 (15.4)	
**BMI, median [IQR]**	26.7 [24.1, 29.9]	26.7 [24.1, 29.9]	26.1 [23.3, 29.1]	<0.001	26.7 [24.1, 29.9]	26.7 [24.1, 29.9]	26.6 [23.9, 29.7]	0.022
**Body fat percentage, n (%)**	31.5 (8.5)	31.5 (8.5)	28.4 (8.4)	<0.001	31.5 (8.5)	31.5 (8.5)	28.8 (8.2)	<0.001
**Impedance of whole body, median [IQR]**	595.0 [533.0, 663.0]	595.0 [533.0, 663.0]	583.0 [526.0, 655.0]	0.060	595.0 [533.0, 663.0]	595.0 [533.0, 663.0]	570.0 [518.0, 643.0]	<0.001
**Whole body fat mass (kg), median [IQR]**	23.3 [18.3, 29.7]	23.3 [18.3, 29.7]	21.3 [16.1, 27.1]	<0.001	23.3 [18.3, 29.7]	23.3 [18.3, 29.7]	22.1 [17.1, 28.0]	<0.001
**Weight change compared with 1 year ago, n (%)**				0.065				<0.001
Yes, gained weight	140742 (28.5)	140578 (28.5)	164 (31.2)		140578 (28.5)	140195 (28.6)	383 (24.6)	
Yes, lost weight	75807 (15.4)	75712 (15.4)	95 (18.1)		75712 (15.4)	75429 (15.4)	283 (18.2)	
**Alcohol drinker status, n (%)**				<0.001				<0.001
Previous	18093 (3.6)	18039 (3.6)	54 (10.1)		18039 (3.6)	17929 (3.6)	110 (6.9)	
Current	460242 (91.8)	459770 (91.8)	472 (88.4)		459770 (91.8)	458340 (91.8)	1430 (90.3)	
Never	22379 (4.5)	22371 (4.5)	8 (1.5)		22371 (4.5)	22329 (4.5)	42 (2.7)	
**Alcohol intake frequency, n (%)**				0.013				<0.001
Daily or almost daily	101746 (20.3)	101621 (20.3)	125 (23.4)		101621 (20.3)	101156 (20.2)	465 (29.4)	
Three or four times a week	115413 (23.0)	115304 (23.0)	109 (20.4)		115304 (23.0)	114987 (23.0)	317 (20.0)	
Once or twice a week	129258 (25.8)	129123 (25.8)	135 (25.3)		129123 (25.8)	128751 (25.8)	372 (23.5)	
One to three times a month	55836 (11.1)	55788 (11.1)	48 (9.0)		55788 (11.1)	55659 (11.1)	129 (8.1)	
Special occasions only	57989 (11.6)	57934 (11.6)	55 (10.3)		57934 (11.6)	57787 (11.6)	147 (9.3)	
Never	40624 (8.1)	40562 (8.1)	62 (11.6)		40562 (8.1)	40409 (8.1)	153 (9.7)	
**Smoking status, n (%)**				<0.001				<0.001
Never	273449 (54.7)	273274 (54.7)	175 (33.2)		273274 (54.7)	272788 (54.8)	486 (30.8)	
Previous	173008 (34.6)	172723 (34.6)	285 (54.1)		172723 (34.6)	172050 (34.5)	673 (42.6)	
Current	52961 (10.6)	52894 (10.6)	67 (12.7)		52894 (10.6)	52475 (10.5)	419 (26.5)	
**IPAQ activity group, n (%)**				0.008				0.001
Low	76190 (15.2)	76086 (15.2)	104 (19.5)		76086 (15.2)	75811 (15.2)	275 (17.3)	
Moderate	163987 (32.6)	163811 (32.6)	176 (33.0)		163811 (32.6)	163353 (32.7)	458 (28.9)	
High	162096 (32.3)	161953 (32.3)	143 (26.8)		161953 (32.3)	161456 (32.3)	497 (31.3)	
**Major dietary changes in the last 5 years, n (%)**				<0.001				<0.001
Yes, because of illness	55517 (11.1)	55304 (11.1)	213 (40.0)		55304 (11.1)	55010 (11.0)	294 (18.6)	
**Processed meat intake, n (%)**				0.003				<0.001
2-4 times a week	135303 (27.0)	135119 (27.0)	184 (34.8)		135119 (27.0)	134616 (27.0)	503 (31.8)	
**Overall health rating, n (%)**				<0.001				<0.001
Poor	22768 (4.6)	22687 (4.5)	81 (15.2)		22687 (4.5)	22548 (4.5)	139 (8.8)	
Fair	105333 (21.1)	105151 (21.1)	182 (34.1)		105151 (21.1)	104704 (21.0)	447 (28.4)	
**Number of treatments medications taken, median [IQR]**	2.0 [0.0, 4.0]	2.0 [0.0, 4.0]	2.0 [1.0, 5.0]	<0.001	2.0 [0.0, 4.0]	2.0 [0.0, 4.0]	2.0 [1.0, 4.0]	<0.001
**Long standing illness, disability or infirmity, n (%)**				<0.001				<0.001
Yes	159854 (32.6)	159560 (32.6)	294 (57.0)		159560 (32.6)	158877 (32.6)	683 (44.1)	
**Vascular heart problems diagnosed by doctor, n (%)**				<0.001				<0.001
High blood pressure	120138 (23.9)	119994 (23.9)	144 (27.0)		119994 (23.9)	119583 (23.9)	411 (25.9)	
**Mouth teeth dental problems, n (%)**				<0.001				<0.001
Bleeding gums	51144 (10.2)	51106 (10.2)	38 (7.1)		51106 (10.2)	50979 (10.2)	127 (8.0)	
Dentures	63443 (12.6)	63315 (12.6)	128 (24.0)		63315 (12.6)	63000 (12.6)	315 (19.8)	
**Pain types experienced in last month, n (%)**				<0.001				<0.001
Facial pain	3533 (0.7)	3504 (0.7)	29 (5.4)		3504 (0.7)	3480 (0.7)	24 (1.5)	
Headache	102938 (20.5)	102846 (20.5)	92 (17.2)		102846 (20.5)	102563 (20.5)	283 (17.8)	
Neck or shoulder pain	73835 (14.7)	73721 (14.7)	114 (21.3)		73721 (14.7)	73468 (14.7)	253 (15.9)	
**Wheeze or whistling in the chest in last year, n (%)**				0.052				<0.001
Yes	103262 (21.0)	103131 (21.0)	131 (25.0)		103131 (21.0)	102691 (21.0)	440 (28.3)	

^a^Chi-square tests were used for categorical variables and t-tests for continuous variables. BMI, body mass index; IPAQ, International Physical Activity Questionnaire; IQR, interquartile range.

Moreover, fewer participants with a history of HNC at baseline were self-reported current alcohol drinkers, and more participants self-reported as previous alcohol drinkers. More participants were also currently tobacco smokers or prior smokers. Moreover, major dietary changes in the last 5 years due to illness were more common. No correlation was found between HNC and ethnic background, weight change compared to the previous year, Metabolic Equivalent Task (MET) min per week for walking, diabetes status, wheezing or whistling in the chest during the past year, or processed meat/fish (oily or non-oily) intake.

### Incident HNC characteristics.

In total, 1,587 of the 501,833 controls (median follow-up: 12 years) at baseline were diagnosed with HNC during the follow-up period (incident cases). The most common HNC diagnoses among the incident cases were malignant neoplasm of tonsil (ICD-10 code C09), malignant neoplasm of larynx (ICD-10 code C32), and malignant neoplasm of base of tongue (ICD-10 code C01). In addition to the clinical variables and coexisting medical conditions associated with an HNC diagnosis at baseline, incident cases were also more likely to drink alcohol (daily or almost daily), smoke most or all days, have wheezing or whistling in the chest during the past year, shortness of breath walking on level ground, a decreased forced expiratory volume in 1 second/forced vital capacity ratio (FEV1/FVC) due to higher FVC (liters), as well as consume processed meat 2-4 times per week (Table 1 and S2–S9 Tables; *P* <  0.001). Compared to HNC cases at baseline, incident cases no longer demonstrated a significant difference in BMI.

Of the 125 SNPs associated with HNC development in the NHGRI-EBI GWAS Catalog, UKB GWAS data were available for 25 (20%). In total, 3 of the 25 (12%) SNPs showed significant differences in genotype frequency between HNC incident cases and controls (Table 2; *P* <  0.001). One SNP had a higher genotype frequency of the major allele homozygous in patients with HNC (rs259919-G [*ZNRD1ASP* gene]), whereas rs1131769-T (*STING1* gene) and rs28419191-T (*ECSCR - SMIM33* gene) were more prevalent in controls.

**Table 2:** GWAS SNPs associated with head and neck cancer, UK Biobank 2006-2021.

	Incidence			
Characteristic	Overall (n = 501833)	No HNC (n = 500246)	HNC (n = 1587)	p
**rs1029239-C (*TRIM15*, 6p22.1), n (%)**				0.006
0	108183 (22.2)	107802 (22.2)	381 (24.9)	
1	242371 (49.8)	241602 (49.8)	769 (50.2)	
2	136197 (28.0)	135816 (28.0)	381 (24.9)	
**rs1051512-C (*TFAP2A*, 6p24.3), n (%)**				0.38
0	260 (0.1)	258 (0.1)	2 (0.1)	
1	22398 (4.6)	22324 (4.6)	74 (4.8)	
2	464494 (95.3)	463041 (95.3)	1453 (95.0)	
**rs10950641-G (*SNX8*, 7p22.3), n (%)**				0.05
0	1333 (0.3)	1324 (0.3)	9 (0.6)	
1	46469 (9.5)	46316 (9.5)	153 (10.0)	
2	439414 (90.2)	438046 (90.2)	1368 (89.4)	
**rs11068315-C (*TESC*, 12q24.22), n (%)**				0.677
0	2828 (0.6)	2819 (0.6)	9 (0.6)	
1	66313 (13.6)	66093 (13.6)	220 (14.4)	
2	417183 (85.8)	415885 (85.8)	1298 (85.0)	
**rs11101731-G (*PAOX*, 10q26.3), n (%)**				0.643
0	2807 (0.6)	2798 (0.6)	9 (0.6)	
1	68489 (14.1)	68261 (14.1)	228 (14.9)	
2	415090 (85.3)	413798 (85.3)	1292 (84.5)	
**rs111332410-C (*SLCO4A1*, 20q13.33), n (%)**				0.792
0	992 (0.2)	990 (0.2)	2 (0.1)	
1	42003 (8.6)	41874 (8.6)	129 (8.4)	
2	443790 (91.2)	442394 (91.2)	1396 (91.4)	
**rs1131769-T (*STING1*, 5q31.2), n (%)**				<0.001
0	370133 (76.2)	369045 (76.2)	1088 (71.2)	
1	107834 (22.2)	107431 (22.2)	403 (26.4)	
2	7857 (1.6)	7820 (1.6)	37 (2.4)	
**rs116168967-G (*LINC00882*, 3q13.11), n (%)**				0.792
0	111 (0.0)	111 (0.0)	0 (0.0)	
1	16048 (3.3)	16000 (3.3)	48 (3.1)	
2	470420 (96.7)	468941 (96.7)	1479 (96.9)	
**rs1229984-T (*ADH1B*, 4q23), n (%)**				0.016
0	457104 (94.1)	455643 (94.1)	1461 (95.7)	
1	26504 (5.5)	26446 (5.5)	58 (3.8)	
2	1970 (0.4)	1963 (0.4)	7 (0.5)	
**rs12433985-A (*SLC7A7*, 14q11.2), n (%)**				0.4
0	67065 (13.8)	66858 (13.8)	207 (13.6)	
1	226471 (46.6)	225784 (46.6)	687 (45.1)	
2	192365 (39.6)	191737 (39.6)	628 (41.3)	
**rs150615-A (*KRT8P4 - RIPK2*, 8q21.3), n (%)**				0.562
0	129297 (26.6)	128906 (26.6)	391 (25.6)	
1	242779 (49.9)	242019 (49.9)	760 (49.8)	
2	114462 (23.5)	114088 (23.5)	374 (24.5)	
**rs174549-G (*FADS1*, *FADS2*, 11q12.2), n (%)**				0.82
0	45279 (9.3)	45131 (9.3)	148 (9.7)	
1	202473 (41.6)	201847 (41.6)	626 (41.0)	
2	238980 (49.1)	238227 (49.1)	753 (49.3)	
**rs17612-T (*C5*, 9q33.2), n (%)**				0.236
0	2334 (0.5)	2326 (0.5)	8 (0.5)	
1	61824 (12.7)	61608 (12.7)	216 (14.1)	
2	422819 (86.8)	421513 (86.8)	1306 (85.4)	
**rs2523608-G (*HLA-B*, 6p21.33), n (%)**				0.048
0	176512 (36.2)	175912 (36.2)	600 (39.2)	
1	232580 (47.8)	231875 (47.8)	705 (46.0)	
2	77864 (16.0)	77638 (16.0)	226 (14.8)	
**rs2571400-C (*HLA-W* - *MICD*, 6p22.1), n (%)**				0.191
0	118797 (24.7)	118397 (24.7)	400 (26.4)	
1	240246 (49.9)	239492 (49.9)	754 (49.7)	
2	122585 (25.5)	122223 (25.5)	362 (23.9)	
**rs259919-G (*ZNRD1ASP*, 6p22.1), n (%)**				<0.001
0	58148 (12.0)	57914 (11.9)	234 (15.3)	
1	218794 (45.0)	218117 (45.0)	677 (44.2)	
2	209561 (43.1)	208942 (43.1)	619 (40.5)	
**rs2641256-A (*SCIMP*, *ZNF594-DT*, 17p13.2), n (%)**				0.27
0	232191 (47.7)	231462 (47.7)	729 (47.6)	
1	203980 (41.9)	203319 (41.9)	661 (43.2)	
2	50184 (10.3)	50044 (10.3)	140 (9.2)	
**rs28419191-T (*ECSCR* - *SMIM33*, 5q31.2), n (%)**				<0.001
0	373746 (76.9)	372651 (77.0)	1095 (71.7)	
1	104776 (21.6)	104375 (21.6)	401 (26.3)	
2	7226 (1.5)	7195 (1.5)	31 (2.0)	
**rs2857595-G (*NCR3* - *UQCRHP1*, 6p21.33), n (%)**				0.777
0	23076 (4.7)	23000 (4.7)	76 (5.0)	
1	163679 (33.6)	163177 (33.6)	502 (32.9)	
2	300005 (61.6)	299056 (61.6)	949 (62.1)	
**rs3823363-C (*HCG9*, 6p22.1), n (%)**				0.005
0	118169 (24.3)	117744 (24.3)	425 (27.9)	
1	242694 (49.9)	241968 (49.9)	726 (47.6)	
2	125195 (25.8)	124820 (25.8)	375 (24.6)	
**rs506770-G (*HSPA1A*, 6p21.33), n (%)**				0.545
0	287610 (59.6)	286694 (59.6)	916 (60.4)	
1	167784 (34.7)	167261 (34.7)	523 (34.5)	
2	27479 (5.7)	27402 (5.7)	77 (5.1)	
**rs55864736-G (*MYO16*, 13q33.3), n (%)**				0.811
0	39368 (8.1)	39251 (8.1)	117 (7.7)	
1	196190 (40.4)	195571 (40.4)	619 (40.5)	
2	250097 (51.5)	249305 (51.5)	792 (51.8)	
**rs692309-T (*DTWD1* - *ATP8B4*, 15q21.2), n (%)**				0.337
0	152150 (31.2)	151648 (31.2)	502 (32.8)	
1	239446 (49.2)	238701 (49.2)	745 (48.7)	
2	95287 (19.6)	95004 (19.6)	283 (18.5)	
**rs75622279-G (*CNTN4*, 3p26.2), n (%)**				0.685
0	1618 (0.3)	1612 (0.3)	6 (0.4)	
1	45464 (9.4)	45330 (9.4)	134 (8.8)	
2	435264 (90.2)	433889 (90.2)	1375 (90.8)	
**rs77045180-G (*AKR1C1*, 10p15.1), n (%)**				0.451
0	175 (0.0)	175 (0.0)	0 (0.0)	
1	17671 (3.6)	17623 (3.6)	48 (3.1)	
2	469317 (96.3)	467837 (96.3)	1480 (96.9)	

^a^Chi-square tests were used for categorical variables. P-value <  0.001 was considered statistically significant. Gene symbols and chromosome band are listed in parentheses for each genome-wide association study (GWAS) single-nucleotide polymorphism (SNP). Genotypes are labeled as 0 =  major allele homozygous, 1 =  heterozygous, and 2 =  minor allele homozygous.

### Relative variable importance and model performance

**Fig 2:**
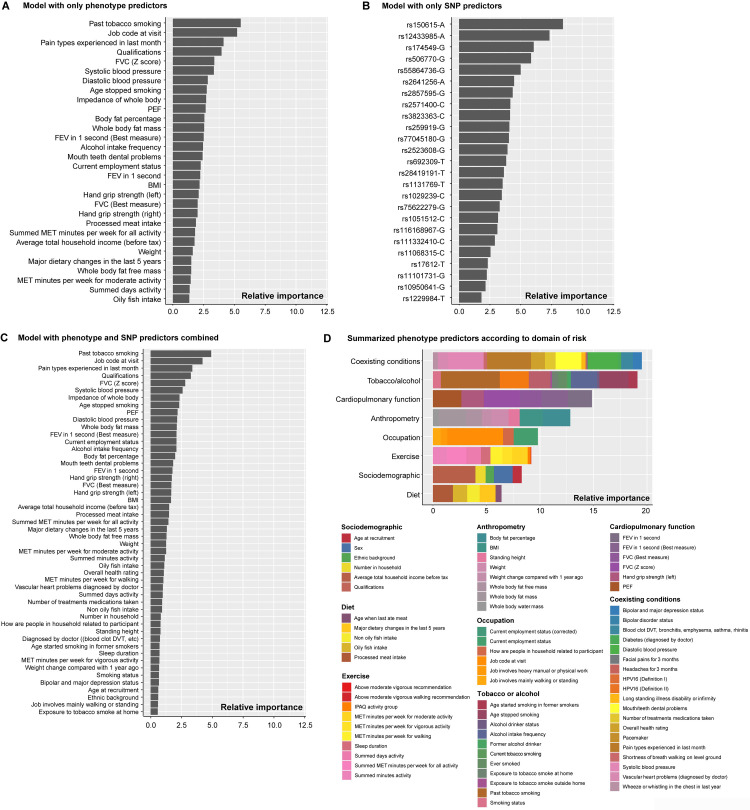
Relative variable importances.

The highest-ranking predictors in the phenotype model included past tobacco smoking, job code at baseline, pain experienced during the last month, qualifications, FVC (Z score), and blood pressure (Fig 2). No substantial differences in relative importance were found among the phenotype predictors, with the exception of smoking and job code at baseline (i.e., the remaining predictors displayed little overall difference in relative importance (Fig 2A). Although rs150615-A (*KRT8P4 - RIPK2* gene), rs12433985-A (*SLC7A7* gene), rs174549-G (*FADS1, FADS2* genes), rs506770-G (*HSPA1A* gene), and rs55864736-G (*MYO16* gene) were identified as the strongest predictors in the SNP model (Fig 2B), none of the 25 SNPs were among the top 50 most important variables in the combined model (Fig 2C).

**Fig 3:**
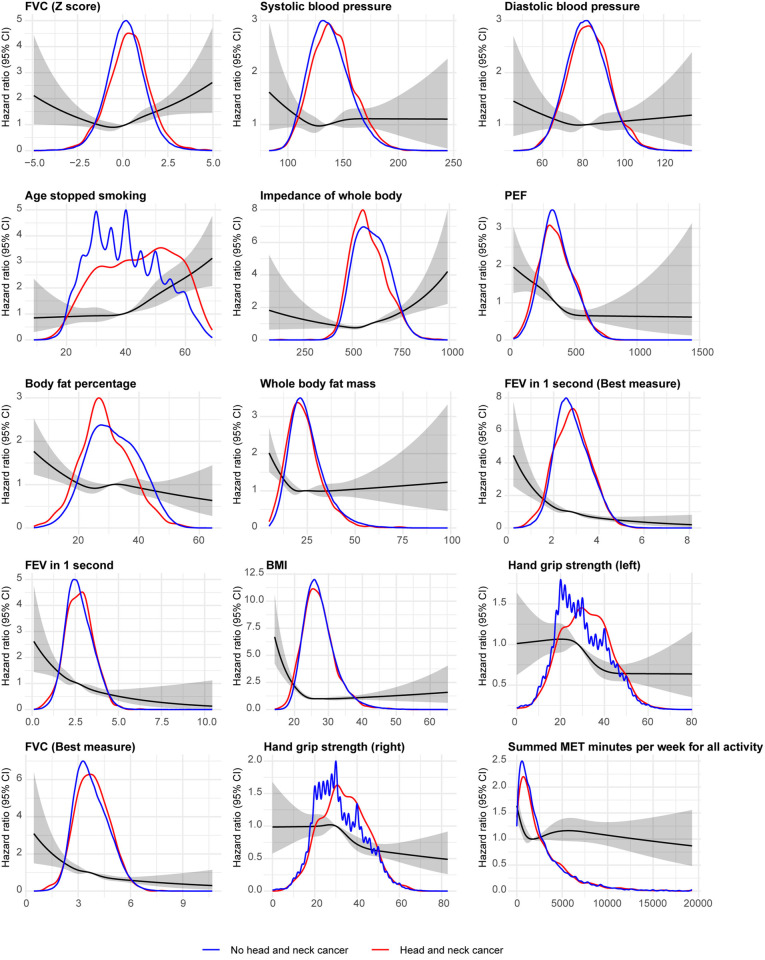
Hazard ratios for continuous variables.

**Fig 4:**
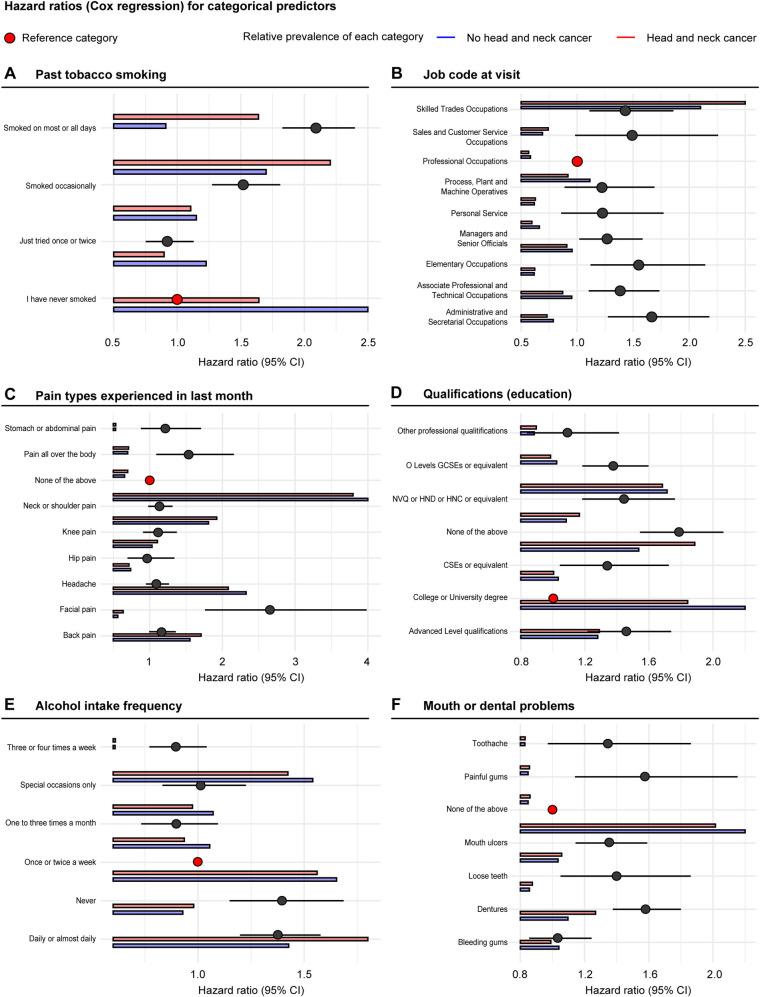
Hazard ratios (Cox Regression) for categorical predictors.

In an evaluation of the relative importance of individual predictors categorized by predictor type (domains of risk), the combined relative importance was dominated by coexisting conditions, tobacco, alcohol, cardiopulmonary function, and anthropometry (Fig 2D). Occupational information, exercise, sociodemographics, and diet displayed lower combined importance. Notably, job code at baseline was the single strongest predictor along with past tobacco smoking. A strong association was noted for age at smoking cessation. A sharp increase in the risk of HNC was shown for individuals that stopped smoking after 40 years of age, suggesting that smoking cessation prior to 40 years of age was not associated with an increased risk of HNC (Fig 3). In contrast, smoking cessation at age 60 was associated with a 2.5-fold increased risk of HNC. Low PEF, low FEV1, and low body fat were also associated with a higher risk of HNC. Increased levels of exercise (MET minutes per week) were associated with a lower risk of HNC. Compared to non-smokers, those who smoked on most or all days displayed a 2-fold hazard ratio of HNC (Fig 4). In comparison with professional occupations, 5 of the 8 job categories displayed a higher risk of HNC, with the most pronounced hazard ratio being noted for those in administrative and secretarial occupations. Compared to participants who drank alcohol once or twice a week, daily drinkers had a 1.25-fold increased risk of HNC, which was similar to those who never drank.

**Fig 5:**
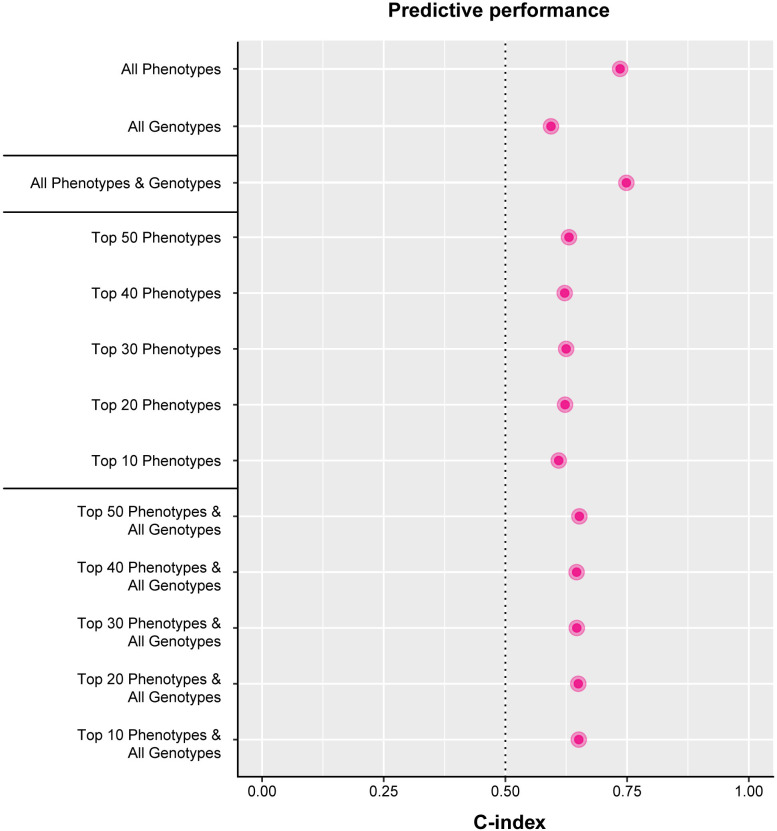
Predictive performance.

The model including all genotypes (SNPs) had a C-index (predictive performance) of 0.60 compared to 0.73 for the model containing all the phenotypic variables (Fig 5). Combining the genotypes and phenotypes only improved the model to 0.75. Using fewer phenotypic variables resulted in poorer model performance.

## Discussion

In the present study, we used genetic, clinical, lifestyle, and sociodemographic data for over 500,000 UK Biobank participants to identify phenotypic features and genetic variants associated with HNC and then evaluated the extent to which these variables could predict incident cases of HNC. We assessed a wide range of predictors in order to evaluate their relative importance for predicting HNC using a state-of-the-art data-driven machine learning model. This allows for an unbiased approach to identifying novel predictors and also reassessing the importance of known risk factors of HNC. Since we only had access to 25 out of 125 SNPs known to be associated with HNC, and the individual effect of a SNP is typically very small, we are surprised that the SNP model achieved a C-index of 0.60, suggesting that these genetic variants are indeed implicated in the development of HNC. We also found that a model including only 10 phenotype predictors achieved a C-index similar to the model including 74 variables. Tobacco smoking, occupation, pulmonary function, and anthropometric measures dominated the phenotype model, with the strongest predictor being modifiable, namely smoking. Interestingly, we found that smoking cessation at 40 years of age or earlier did not confer a risk of HNC, such that ex-smokers had an elevated risk of HNC if smoking cessation occurred after 40 years of age. However, this study cannot further stratify this potential risk reduction temporally. In addition, the model identified similar relative risk for HNC among the participants who registered daily alcohol consumption as the group registered as never consuming alcohol, which is a surprising finding as alcohol consumption is a previously known risk factor. Speculatively, this could be caused by misinterpretation of the available categories upon registration.

It is important to note that while the SNP model was slightly better than chance, none of the 25 SNPs were among the strongest predictors of HNC in the combined model, indicating that genetic variants convey little modification of risk. However, it should be emphasized that a C-index of 0.60 is significant for a model containing only genetic variants. Moreover, the full phenotype model had a C-index of 0.73, suggesting a clinically meaningful prediction model [32]. This model was dominated by variables that are readily available in most clinical settings (i.e., history of tobacco smoking, occupation, pulmonary function, and anthropometric measures). It is however possible that the effect of the genetic component could be underexaggerated as this study could only access 20% (25/125) of the identified SNPs with connections to HNC development. Future research endeavors should make efforts to widen the genetic scope, to ensure that a more representative conclusion can be drawn, perhaps through combination of different databases or through conducting more extensive testing.

Smoking cessation around the time of an HNC diagnosis has been associated with improved outcomes, i.e., more favorable survival rates [33], better treatment response [34], and a lower risk of recurrence [35]. Despite motivation to quit smoking, newly diagnosed HNC patients are rarely able to abstain longer than 30 days [36]. In a recent prospective study, Van Heest *et al.* showed that approximately 60% of surviving patients with HNC continued to be active smokers 6-24 months after treatment, with successful smoking cessation most likely to occur within the first 6 months of treatment start [37]. However, few studies investigating the impact of smoking cessation on HNC included patients under the age of 50 or 60 [38]. Therefore, studies to confirm the effect of age and smoking cessation on HNC risk are warranted.

Exposure to certain substances such as asbestos and wood dust are known risk factors for larynx- and sinonasal cancer, respectively [39]. In a large-scale study, the International Head and Neck Cancer Epidemiology (INHANCE) consortium recently demonstrated an increasing risk of HNC for patients with increasing time employed as service workers, production workers, transport equipment operators, and laborers [39]. These findings are in line with the results of the present study, where we also show that sedentary occupations (e.g., administrative and secretarial jobs) have the highest risk of HNC among the tested job codes in the UKB. When comparing this finding to past literature where varied associations between physical activity and HNC have been reported [40,41], this seems to be a novel finding.

UKB is a large-scale prospective cohort study with baseline and follow-up health-related data and genome-wide genotyping data for over 500,000 participants recruited from around the United Kingdom [42]. In the present study, the UKB cohort had a median follow-up of approximately 12 years with 0.4% of participants reported as having an ICD-10 code associated with HNC at baseline or after follow-up. In line with GLOBOCAN 2020 data, malignant neoplasms of the tonsil, larynx, and base of tongue were the most common HNC diagnoses [2]. Given that HPV16 seropositivity testing was only performed in a pilot study containing 10,000 UKB participants (excluding individuals with potentially HPV-associated malignancies), it was not possible to evaluate HPV in the present study in a statistically sufficient manner [43]. Due to the voluntary nature of study enrollment in the UKB, cancer rates in the UKB may be below that of the general population. This may be due to selective inclusion of individuals leading healthy lifestyles and with higher socioeconomic status [42]. However, while the distribution of lifestyle factors, socioeconomic status, and coexisting conditions, may differ in the UKB from the general population, the dataset is large enough to include individuals from all categories, allowing for comparisons to be made.

To our knowledge, relatively few studies have used UKB data (as a training or validation cohort) for HNC risk prediction using phenotypic characteristics [44] or phenotypic characteristics combined with genetic variants [18]. In contrast to the present study that used multivariate Cox regression and machine learning, the studies by McCarthy and Budhathoki used logistic regression to estimate HNC risk. In addition to including genetic variants as well as phenotypic characteristics, the present study includes updated information on reported HNC incidence 5 years after the McCarthy study. The Budhathoki study developed risk models using phenotypic characteristics combined with polygenic risk scores for HNC (containing 22 genetic variants), and separately for cancers of the oral cavity and oropharynx [18]. Although logistic regression is an efficient framework, it makes strong assumptions about the linear relationship between the outcome and each predictor. Non-linear associations, multicollinearity, interactions and large complex models (with many predictors) are difficult to optimize using logistic regression. These obstacles are overcome with gradient boosting, which almost ubiquitously outperforms any regression model for classification and regression.

To the best of our knowledge, this is the largest cohort study (with over a decade follow-up) to date investigating HNC risk prediction using both phenotypic and genotyping data. By evaluating the relative importance or contribution of the inputted variables (phenotypes and SNPs) to the prediction model, we were able to show that phenotypic characteristics contribute to the model significantly more than genotyping data [45]. Our study findings need to be validated using other populations with large-scale studies, e.g., FinnGen [46] and BioBank Japan Project [47].

## Conclusion

In conclusion, this study highlights the utility of phenotypic predictors, including past tobacco smoking habits, occupation, facial pain, education, pulmonary function, and anthropometric measures, in predicting the risk of HNC. While the inclusion of 20% of known SNPs contribute to a better model than chance, their inclusion did not significantly enhance the predictive accuracy compared to models based solely on phenotypic variables. Importantly, the study underscores the protective effect of smoking cessation, particularly when achieved before the age of 40, to eradicate the increased risk of head and neck cancer caused by smoking. These findings emphasize the value of comprehensive phenotypic risk assessment and the potential for early identification and intervention in at-risk populations, thereby informing public health strategies and clinical decision-making for HNC prevention.

## Supporting information

S1 Table Single-nucleotide polymorphisms (SNPs) associated with head and neck cancer according to GWAS Catalog. (XLSX)

S2 Table Sociodemographic variables associated with head and neck cancer, UK Biobank 2006-2021. (XLSX)

S3 Table Anthropometry associated with head and neck cancer, UK Biobank 2006-2021. (XLSX)

S4 Table Characteristics of cardiopulmonary function associated with head and neck cancer, UK Biobank 2006-2021. (XLSX)

S5 Table Coexisting conditions associated with head and neck cancer, UK Biobank 2006-2021. (XLSX)

S6 Table Dietary variables associated with head and neck cancer, UK Biobank 2006-2021. (XLSX)

S7 Table Exercise variables associated with head and neck cancer, UK Biobank 2006-2021. (XLSX)

S8 Table Occupational variables associated with head and neck cancer, UK Biobank 2006-2021. (XLSX)

S9 Table Tobacco and alcohol variables associated with head and neck cancer, UK Biobank 2006-2021. (XLSX)
