# Correction: Genetic, clinical, lifestyle and sociodemographic risk factors for head and neck cancer: A UK Biobank study

**DOI:** 10.1371/journal.pone.0342039

**Published:** 2026-02-02

**Authors:** The PLOS One Editors

## Notice of Republication

This article [1] was republished on December 22, 2025, to address an issue identified post-publication. Please download this article again to view the correct version.
